# Step by step: Floral structure and developmental changes to the formation of the gynostegium in Apocynaceae s.l.

**DOI:** 10.1111/plb.70225

**Published:** 2026-05-14

**Authors:** D. M. Alves, L. S. Souto, J. L. S. Mayer, I. Koch

**Affiliations:** ^1^ Departamento de Biologia Vegetal Instituto de Biologia, Universidade Estadual de Campinas Campinas São Paulo Brazil; ^2^ Laboratório de Diversidade Vegetal–Departamento de Biologia Universidade Federal de São Carlos, Rodovia João Leme dos Santos (SP‐264) Sorocaba São Paulo Brazil

**Keywords:** Adnation, conation, floral anatomy, floral architecture, floral ontogenesis

## Abstract

- The gynostegium is a key floral innovation in Apocynaceae, formed by the fusion between anthers and the style head and is essential for pollination. We investigated how this complex structure develops by comparing early‐ and late‐divergent species to elucidate the developmental processes underlying its formation and diversification.
- Floral buds and flowers were analysed using light microscopy and scanning electron microscopy, allowing detailed examination of morphological and anatomical traits throughout floral ontogeny.
- We identified the timing and sequence of conation, adnation and fusion events during development. All species exhibited petal union during floral ontogeny, generally consistent with postgenital fusion, although in *Asclepias curassavica*, this process occurs at an earlier developmental stage and shows a distinct ontogenetic pattern. All species also showed adnation of staminal filaments to the corolla tube. We documented progressive stamen specializations, including the formation of a staminal tube, sclerenchymatous differentiation in the anthers, secretory tissues presentation and guide rails, particularly in the APSA clade.
- Our findings demonstrate developmental steps that extend beyond anther–style head fusion. We show that reproductive structures are reshaped, both functionally and anatomically, towards gynostegium formation. The reduction of the corolla tube and emergence of a staminal tube suggest a shift in pollinator‐guiding roles from the corolla to the androecium, accompanied by a partial transfer of secretory functions from the gynoecium to the stamens. These results provide new insights into the developmental mechanisms driving floral complexity in Apocynaceae.

## Graphical abstract

Developmental changes in Apocynaceae s.l. reveal progressive reductions in the corolla tube and epipetaly, together with increased staminal tube formation, highlighting shifts in floral integration associated with gynostegium evolution and organization.

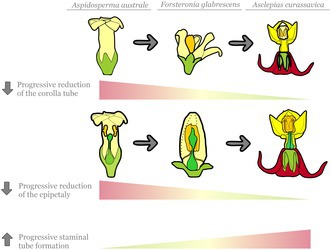

## INTRODUCTION

The Apocynaceae family is among the most diverse angiosperms, comprising 5,350 species across 378 genera (Endress *et al*. 2019). This family is widely distributed in tropical and subtropical regions, with notable diversity in tropical areas such as tropical America, southern Africa and southeast Asia (Ollerton *et al*. 2019; Bitencourt *et al*. 2021). It is well known for supporting a variety of species such as periwinkles (*Vinca* spp.), dogbanes (*Apocynum* spp.) and milkweeds (*Asclepias* spp.). It consists of a wide variety of habits, including almost all growth forms, from small and large trees to scramblers, shrubs, vines, herbs, epiphytes and others (Fishbein *et al*. 2018; Ollerton *et al*. 2019; Bitencourt *et al*. 2021). Such diversity is also reflected in their reproductive characteristics. The morphological variation of their flowers, along with the formation of a unique floral structure in part of the family, the gynostegium, makes Apocynaceae quite interesting, especially from the perspective of floral development (Fallen 1986; Endress & Bruyns 2000; Endress *et al*. 2014; Endress 2016; Fishbein *et al*. 2018; Koch *et al*. 2018). In general terms, the flowers of the family exhibit several distinctive features: they are pentamerous, with a sympetalous corolla; the stamens are adnate to the corolla tube, inserted opposite the sepals and alternating with the petals, with the anthers positioned above or at the level of the style head, two carpels, which may be partially fused at the base of the ovary or by the style, nectaries at the base of the ovary, in the carpel wall or as a distinct structure (rauvolfioid and apocynoid grades) or along the anthers sides, in the guide rails region. A progressive adnation is observed between the stamens and the style head, making it possible to find flowers in which these whorls are entirely free from each other, as observed in the groups that diverge first in the family (rauvolfioid grade), to flowers in which these whorls are intimately united, as seen in the more recently diversified groups (apocynoid grade + Periplocoideae + Secamonoideae + Asclepiadoideae, the APSA clade–Fallen 1986; Endress & Bruyns 2000; Endress 2016; Fishbein *et al*. 2018; Endress *et al*. 2019).

The progressive synorganization between the androecium and the gynoecium to form a structurally and functionally complex unit, the gynostegium, has been considered the major evolutionary trend within the family (Fallen 1986; Simões *et al*. 2007; Endress 2016; Fishbein *et al*. 2018). This floral configuration first emerged in the ancestor of the APSA clade and has since been maintained in most groups, and this complex structure is closely linked to pollination and the dispersal mechanism of pollen grains (Fallen 1986; Kunze 1991; Fishbein 2001; Fishbein *et al*. 2018). In the typically salverform corolla flowers of rauvolfioid grade, this arrangement is guided by a floral tube; the position of the anthers contributes to this arrangement. Usually, these flowers possess nectaries at the base of the ovaries, and access for pollinator proboscis is confined to the spaces between the corolla tube and the gaps between the anthers (Fallen 1986; Lopes & Machado 1999). In apocynoid grade flowers, with their various corolla forms, the anthers exhibit partial adnation to the style head by the connective (Fallen 1986; Simões *et al*. 2007), and the extension of the stamen adnation to the corolla tube is variable. The corolla tube and the anthers direct the insect proboscis. Additionally, a viscous secretion, like glue, is present on the lower portion of the style head (Fallen 1986; Endress 2016). This secretion facilitates the passage of the pollinator proboscis, aiding in the attachment of available pollen grains for pollination at the style head apex (Fallen 1986; Simões *et al*. 2007). In the flower of Asclepiadoideae, the corolla can be variable in form; the anthers are strongly adnate to the style head by cell–cell union or by secretion, and the stamens are described as entirely free from the petals (Kunze 1991; Endress & Bruyns 2000; Fishbein 2001). This arrangement guides pollinator access to the pollinia, which transport pollen grains in aggregated form during the pollination process (Kunze 1991; Fishbein 2001; Endress *et al*. 2014). The emergence of aggregated pollen dispersion, in the form of pollinaria and pollinia, may have played a pivotal role in the diversification of this group. The species with dispersal pollen aggregation, previously referred to as Asclepiadaceae (comprising the subfamilies Periplocoideae, Secamonoideae and Asclepiadoideae), collectively encompasses approximately 3,400 species, making it the most diverse clade within the family Apocynaceae (Meve 2002; Endress 2016; Bitencourt *et al*. 2021).

Despite the significant ecological role of the gynostegium, which supports a complex and efficient pollination system, we cannot infer about the development of the floral structure. In groups where the union between the anthers and the style head begins to form, this process occurs through secretion from the style head (Demeter 1922; Fallen 1986; Simões *et al*. 2007; Demarco 2014, 2017a; 2017b; Monteiro & Demarco 2017). Additionally, aspects related to the secretory structures in the whorls that comprise the gynostegium have been investigated, including nectar secretion and the secretions that form the pollinarium apparatus (Demarco 2014, 2017a, 2017b; Monteiro & Demarco 2017). However, the developmental aspects and the fusion of structures across different floral types remain poorly explored from a comparative perspective.

In this context, our study aims to understand the progressive morphological changes during floral development across distinct flower types that culminate in the formation of the gynostegium structure in the APSA clade (except Periplocoideae and Secamonoideae; see Section 2). This comparative approach, which has not yet been explored in the context of the Apocynaceae family circumscription as currently defined, may provide valuable insights into developmental trends of floral structures. To achieve this goal, we selected three floral types that represent a significant morphological variation within the family and conducted a comparative analysis of their ontogeny: *Aspidosperma australe* Müll. Arg (rauvolfioid grade) represents the lineage that diverges first within the family, with a pronounced corolla tube and included stamens, free from the style head. *Forsteronia glabrescens* Müll. Arg. (apocynoid group) represents an intermediate lineage within the family, characterized by a rotated corolla and exerted stamens and a style head united in a gynostegium. Finally, *Asclepias curassavica* L. (Asclepiadoideae), with campanulate corolla and exerted stamen, presents a gynostegium with pollinarium and well‐pronounced staminal coronas.

## MATERIAL AND METHODS

The three Apocynaceae species studied occur naturally in Brazil. *Aspidosperma australe* has an arboreal habit and is distributed in the South, Southeast and Central‐West regions of Brazil. The vegetational types in which it occurs include Cerrado, Gallery Forest, Semideciduous Seasonal Forest and Ombrophilous Forest. *Forsteronia glabrescens* Müll. Arg. are lianas and climbers with distribution in the South, Southeast, Central‐West and Northeast regions of Brazil. They are found in different vegetational types, including Cerrado, Gallery Forest and Semideciduous Seasonal Forest. *Asclepias curassavica* L. is a terrestrial herb, with distribution covering all Brazilian regions (South, Southeast, Central‐West, North and Northeast). They occur in practically all types of vegetational formations, including anthropized areas, Caatinga (stricto sensu), Cerrado (lato sensu), Gallery Forest, Terra Firme Forest, Deciduous Seasonal Forest, Semideciduous Seasonal Forest, Ombrophilous Forest (Rainforest), Mixed Ombrophilous Forest and Restinga (The Brazil Flora Group *et al*. 2022). We obtained specimens of *A. curassavica* and *F. glabrescens* from individuals occurring in natural populations and *A. australe* from a cultivated individual. The vouchers containing the collected material were deposited in the UEC Herbarium (UNICAMP) under the respective accession numbers: *Aspidosperma australe* (UEC142027), *Forsteronia glabrescens* (UEC208323) and *Asclepias curassavica* (UEC208328).

For anatomical analysis, we collected buds at several developmental stages and anthetic flowers in the field from at least two individuals per species. The samples were fixed in buffered formalin (Clark 1981), dehydrated in an ethanol series, embedded in (2‐hydroxyethyl)‐methacrylate LeicaTM according to the manufacturer's instructions, and transversely and longitudinally sectioned (5–8 μm thick) with a Leica RM 2245 rotary microtome. Sections were stained with toluidine blue (0.05%) in pH 4.7 sodium acetate buffer (O'Brien *et al*. 1964, modified), mounted with water and photomicrographs were taken with an Olympus BX51 photomicroscope.

For surface analysis, the samples were dehydrated in an ethanol series, dissected and critical point‐dried in a Balzers CPD 03 dryer. They were then mounted on aluminium stubs with colloidal carbon and coated with gold using a Balzers Sputter SCD‐050 sputter coater. Samples were observed using a JEOL JSM 5800LV scanning electron microscope. The SEM images were subsequently artificially colourized using Adobe Photoshop software.

## RESULTS

### Floral organography

**Fig. 1:**
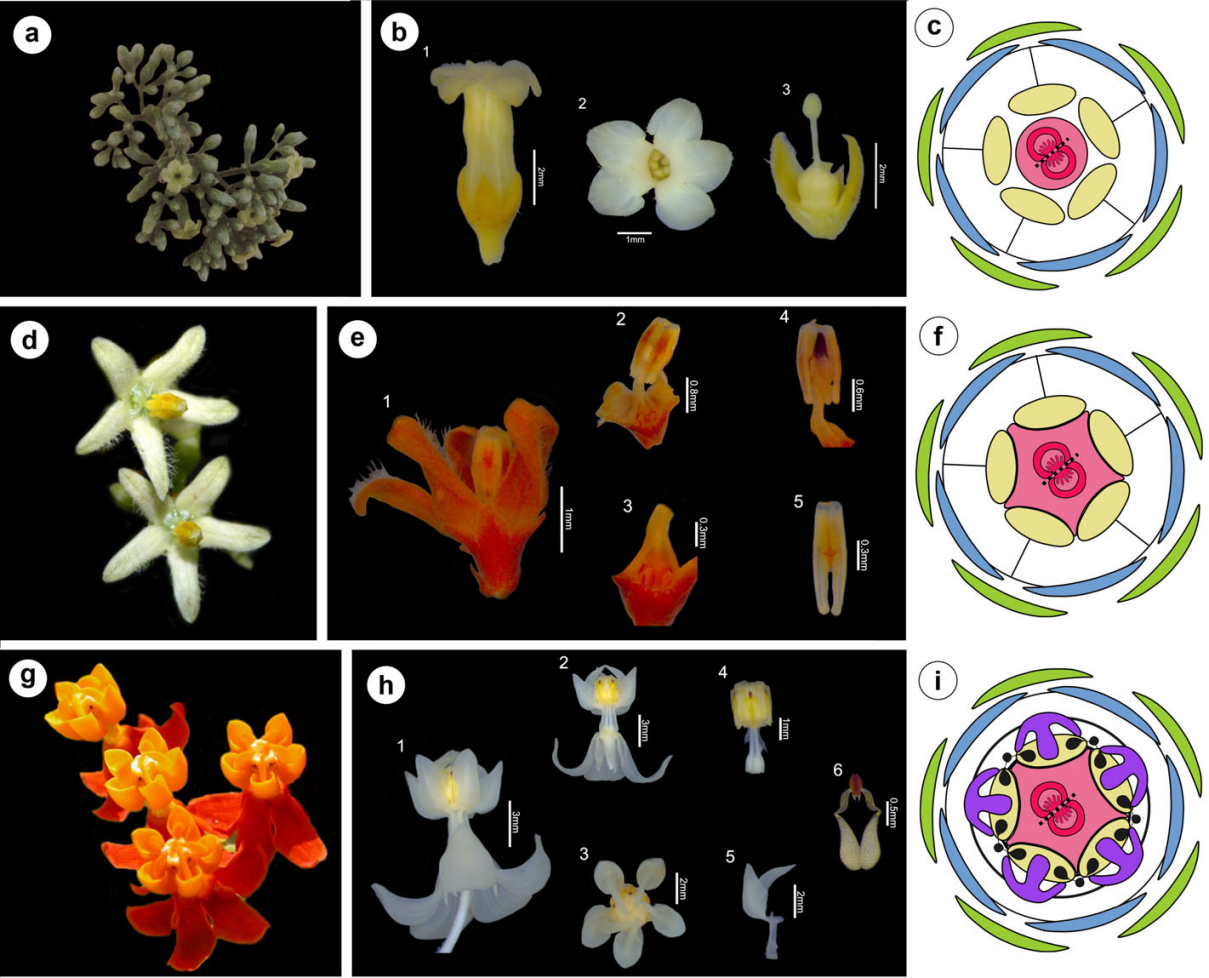
Floral organization of (a–c) *Aspidosperma australe* Mull. Arg. (d–f) *Forsteronia glabrescens* Mull. Arg, and (g–i) *Asclepias curassavica* L. (a, d, g) Flowers in anthesis, (b, e, h) detailed view of flowers under a stereomicroscope (c, f, i) and floral diagrams showing sepals (in green), petals (in blue), anthers (in yellow), style head (in light pink), carpels (in dark pink), gynostegial coronas (in purple) and pollinarium (in black).

*Aspidosperma australe* (Fig. 1a–c), *Forsteronia glabrescens* (Fig. 1d–f) and *Asclepias curassavica* (Fig. 1g–i) have perfect, pentamerous flowers composed of five sepals, five petals forming a sympetalous corolla, five stamens and a bicarpellate gynoecium with a superior ovary. In *A. australe*, the sepals are free. The corolla forms a well‐developed tube, with petals united along most of their length and short free lobes distally. The stamens are adnate to the corolla tube, with only a small distal portion of the filaments remaining free. The anthers are positioned above the style head and remain free from it. The styles are united along most of their length, forming a common style head, while the ovaries are partially united at the apex. In *F. glabrescens*, the sepals are free and the corolla is sympetalous, forming a short tube with elongated free lobes. The stamens are inserted at the base of the corolla tube and are closely arranged around the style. The anthers are united to the style head, forming a gynostegium. The styles are united along their length, and the ovary is superior with partial apical union. In *A. curassavica*, the sepals are free, and the corolla is sympetalous, with a short basal tube and reflexed lobes. The stamens are united to each other, forming a staminal tube surrounding the ovary and style. The anthers are firmly united to the style head, forming a highly integrated gynostegium with pollinaria. The styles are free along most of their length but converge at the style head. The carpels are united only at the base.

### Early developmental stages of *Aspidosperma, Forsteronia* and *Asclepias* species

**Fig. 2:**
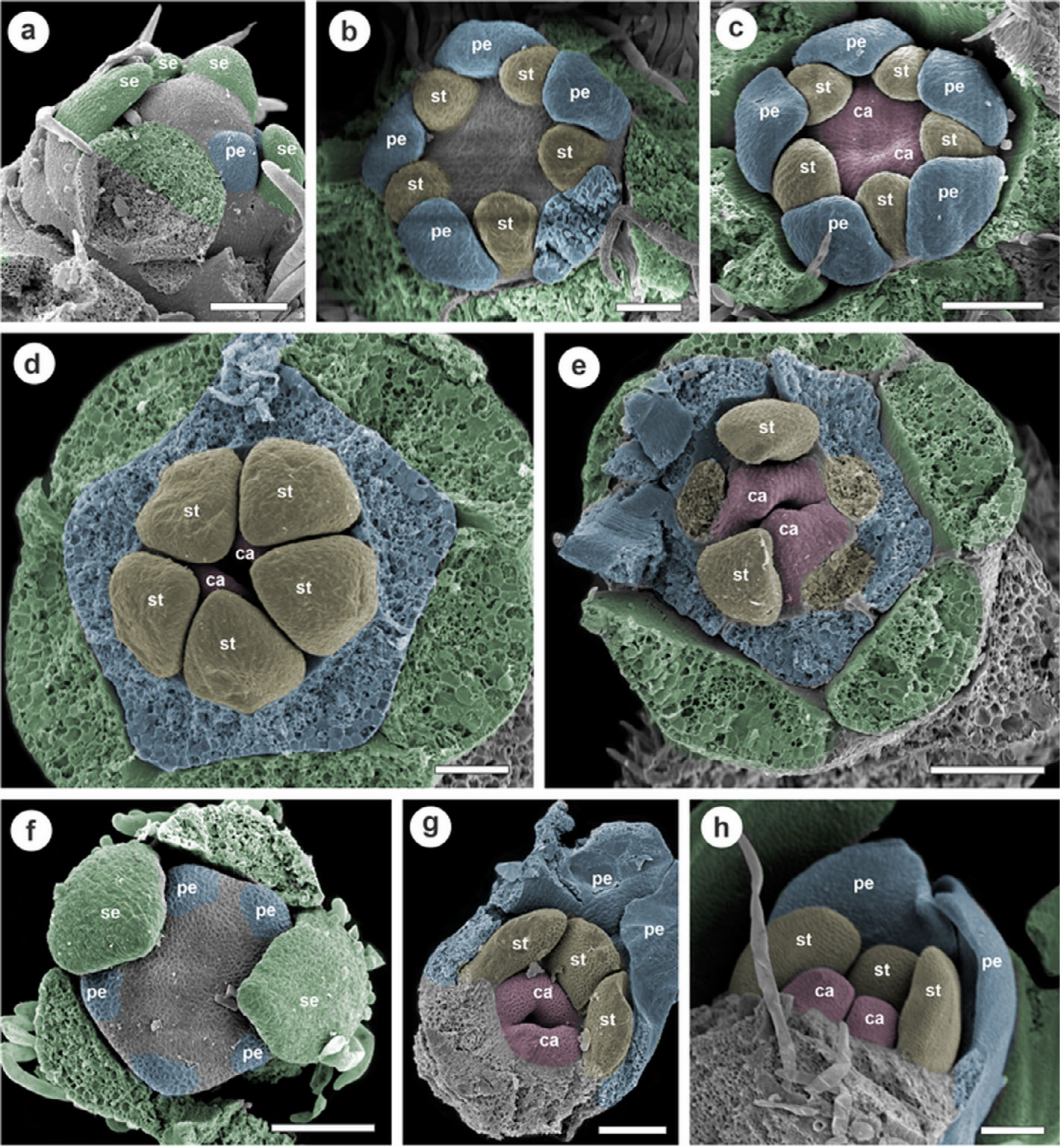
Floral development of (a–c) *Aspidosperma australe* Mull. Arg. (d, e) *Forsteronia glabrescens* Mull. Arg. and (f–h) *Asclepias curassavica* L. under scanning electron microscopy. (a) Young, with established sepals and two petal primordia. (b) Floral primordium with established sepals, petals and stamen primordia. (c) Young floral bud with established sepals, petals, stamen and two carpel primordia. (d) Young floral bud, showing elongating stamens. (e) Carpels elongation. (f) Sepals and the beginning of five petal primordia. (g) Young bud with whorls established. (h) Elongation of the organs. Calix (green); corolla (blue); androecium (yellow), gynoecium (pink); ca: carpel; pe: petal; se: sepal; st: stamen. Scale: A, C, E–H = 100 μm; B, D = 50 μm.

The order of initiation of the floral whorls in all analysed species (*A. australe*, *F. glabrescens* and *A. curassavica*) is centripetal, beginning with the outermost whorl (sepals), followed by petals, stamens and carpels (Fig. 2a–h). In all species, the five sepals and five petal primordia arise as discrete lateral flanks, clearly separated from each other at the time of initiation (Fig. 2a,f). At the stages analysed, no continuous annular meristematic ring was observed at the base of the petal primordia. The corolla tube becomes evident only at later stages of development, after the emergence of the stamen primordia (Fig. 2d,g,h). Following petal initiation, five stamen primordia become visible in an internal position (Fig. 2b,d,g), along with the initiation of two individualized carpel primordia at the centre of the floral meristem (Fig. 2c,g). In *A. australe* and *F. glabrescens*, the floral primordium is conical, and the sepals and petals initiate separately and alternately, showing slight differences in size at early developmental stages (Fig. 2a). In contrast, in *A. curassavica*, the floral primordium is flattened, and sepals and petals emerge nearly simultaneously (Fig. 2f). In all species, the androecium and gynoecium arise as individualized primordia and remain free from each other during the initial stages of development.

### The mid and final developmental stages of the flower of *Aspidosperma australe*

**Fig. 3:**
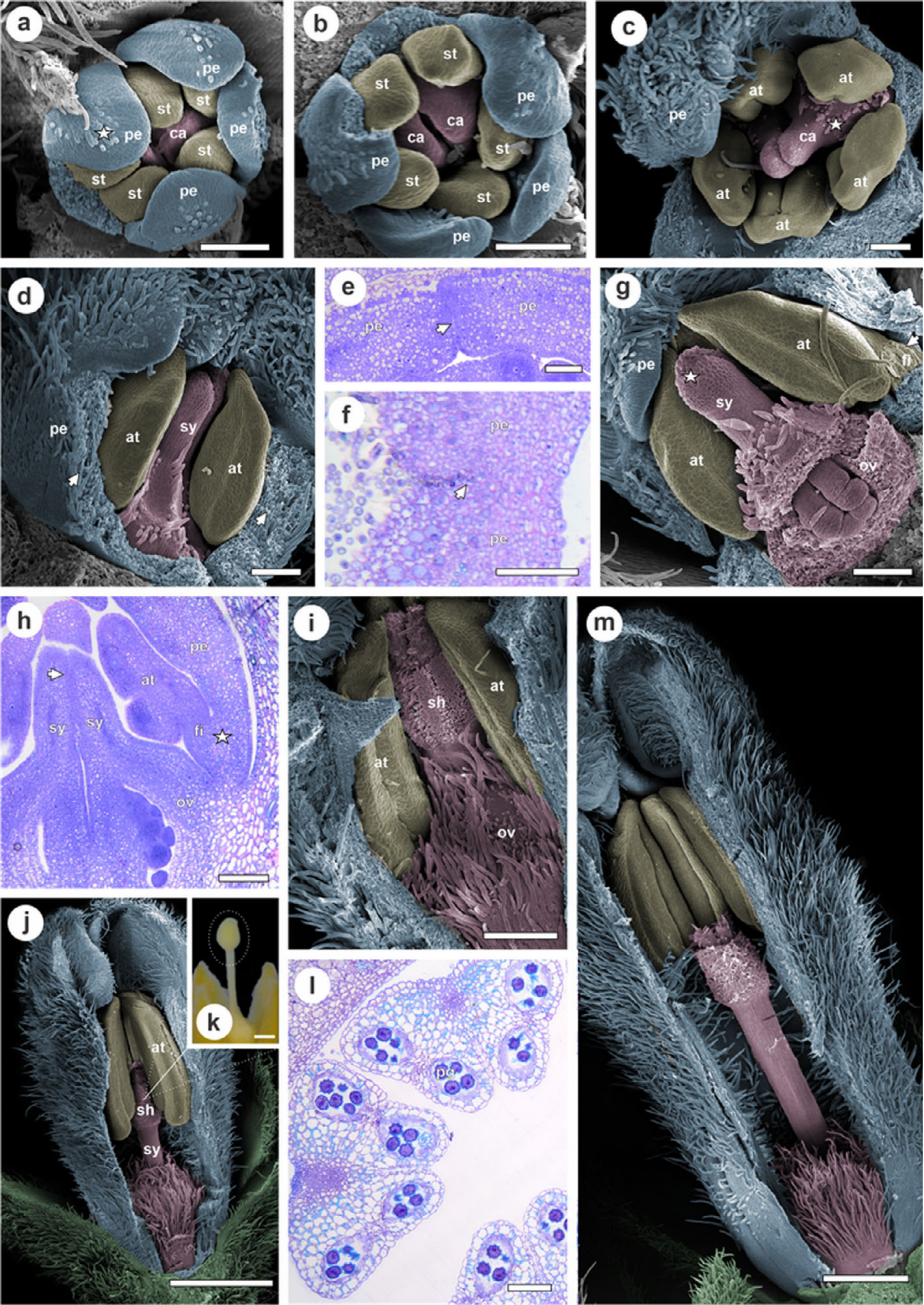
Mid and final developmental stages of *Aspidosperma australe* Mull. Arg. under light (e, f, h, k, l) and scanning electron microscopy (a–d, g, i, j, m). (a) Young bud with stamens and carpels at similar heights and the corolla covering them. (b) Apices of the carpels come together. (c) Petals are covered by indumentum, and anthers are differentiated within the stamens. (d) The corolla tube has a broken fusion part (white arrow) to reveal the fused and free corolla lobes. (e) Corolla tube union detail (white arrow). (f) Union of the corolla tube in enlargement. (g) Ovary region differentiated, filaments of the stamens become epipetalous (white arrow). (h) adnation between filaments and petals (white star) and between the apices of the styles (white arrow). (i) Ovary was completely pilose, and the style head region differentiated. (j) Floral bud before the last elongation. (k) Detail of spool‐shaped style head tips, dotted circle. (l) Mature anthers with microspores developed. (m) Floral bud at the pre‐anthesis stage. Calyx (green); corolla (blue); androecium (yellow); gynoecium (pink); at: anther; ca: carpel; fi: filament; ov: ovary; pe: petal; pg: pollen grains; se: sepal; sh: style head; st: stamen; sy: style. Scale: A–D, F–H, L = 100 μm; E = 50 μm; I = 200 μm; J = 1 mm; K = 0,5 mm; and M = 500 μm.

Since primordia whorls were established, the organs became elongated. Petals became flattened dorsoventrally and enlarged laterally, and the lateral margins of petals became closer to each other and exceeded the stamens in height (Fig. 3a). Stamens and petals elongated and became higher than carpels. At this stage, the petals cover the stamens and acquire indumentum (hairs) on the abaxial surface (Fig. 3a,b). The stamens undergo dorsoventral flattening (Fig. 3b). Subsequently, we can observe that the anthers are the initial part to differentiate in the stamens (Fig. 3c), the apex of carpels becomes closer to each other (Fig. 3b,c) and the proximal region of the carpels acquires hairs (Fig. 3c). The adaxial face of petals acquires indumentum, and the dorsal face of petals turns completely indumented (Fig. 3c). The carpel apex became thinner, forming the styles (Fig. 3d). The lateral margins of the petals are conated, forming a cell‐to‐cell union through the epidermis (Fig. 3d–f), creating the corolla tube. In contrast, the petal apices remain free from each other, characterizing the corolla lobes. The cells at the apex of the styles acquire a global shape, and this region becomes differentiated into the style head. The proximal region is differentiated in the ovary (Fig. 3g). The filaments become differentiated and join to the corolla tube, forming the stamen–corolla tube (sensu Erbar & Leins 1996), and the filament insertion to the anthers is dorsifixed (Fig. 3g–h). At this stage, it is possible to observe the union of the styles (Fig. 3h). After the union of the style's tips, this region elongates to form a spool‐shaped structure (Fig. 3i). The style tips, in conjunction with the spool‐shaped structure (Fig. 3j,k), form the style head region. The ovary region became completely pilose, and the styles underwent another elongation (Fig. 3i). The corolla tube elongates, putting the anthers up to the style head (Fig. 3j) into the pre‐anthesis stage, and both abaxial and adaxial surfaces of the corolla tube become completely pilose (Fig. 3j,m). The anthers have two thecae and four pollen sacs (Fig. 3l) and have no anatomical specializations. During the pre‐anthesis stage, the anthers surpass the style head in height, with the apexes of the styles restricted to the proximal region of the anthers (Fig. 3m).

### The mid and final developmental stages of the flower of *Forsteronia glabrescens*

**Fig. 4:**
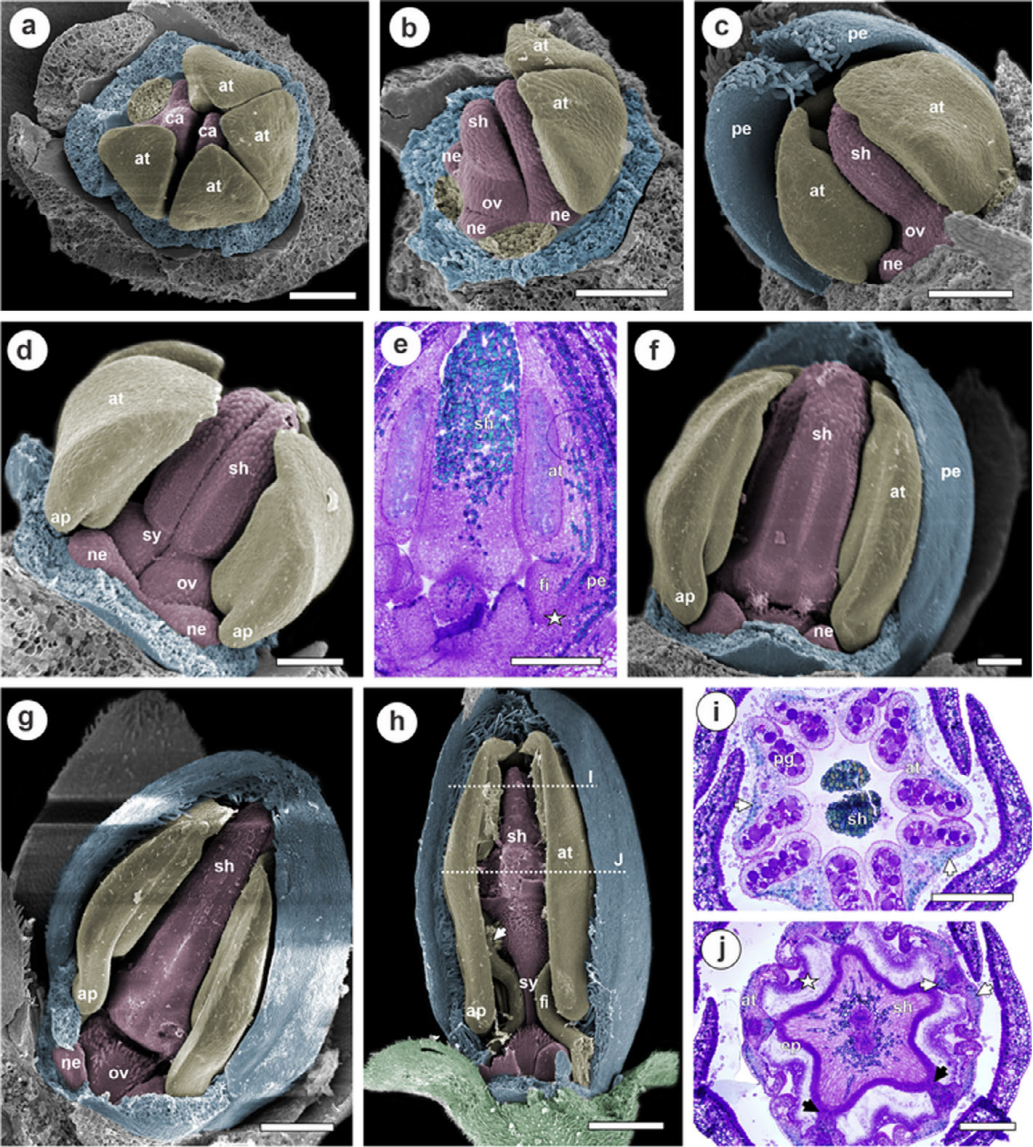
Mid and final developmental stages of *Forsteronia glabrescens* Mull. Arg. under light (e, i, j) and scanning electron microscopy (a–d, f–h). (a) Young bud with stamens and carpels. (b) Differentiation of the carpels into styles and the ovary region with nectaries at the base of the ovary. (c) Stage of whorls elongation. (d) style head, style and ovaries in detail. (e) Filaments are united with the petals (white star) and the cells of the style head with phenolic compounds. (f) Elongation of the basal appendages of the anther. (g) The apex of the style head becomes sharpened. (h) Elongation of the lower part of the style; filaments are inserted ventrally (white arrow). (i) Detail of the upper part of the style head, with the bifid apex; anthers with pollen sacs and sclerenchymatic cells around the connective (white arrow); pollen sacs with monads of pollen grains. (j) The lower part of the style head, showing the expanded portion with epithelial secretory cells and secretion around it (white star); the anthers have sclerenchymatic tissue on the ventral and dorsal sides around the connective (white arrow); there is a union between the epithelium of the style head and the ventral part of the anther (black arrow). Calyx (green); corolla (blue); androecium (yellow), gynoecium (pink); ap: anther basal appendages; at: anther; ca: carpel; fi: filament; ne: nectary; ov: ovary; pe: petal; pg: pollen grains; se: sepal; sh: style head; st: stamen; sy: style. Scale: A–D, F = 100 μm; E, G, I–J = 200 μm; H = 500 μm.

After the initiation of the whorls, the stamen became flattened dorsoventrally, showing the distal region differentiated in the anthers and the proximal region in the filaments (Fig. 4a). The margins of the petals are conated. The corolla tube is visible at the same time the carpels elongate (Fig. 4b,f), the distal region turned dilated with the differentiation of the style head, and the proximal region turned dilated too, with the differentiation in the ovary region (Fig. 4b). Five nectaries are found around the ovary in an antepetalous position (Fig. 4b,c). The apex of the anthers becomes flattened in relation to the pollen sacs portion, and the apex of the petals acquires hairs in the dorsal face (Fig. 4c). The region between the style head and ovary become the narrower beginning of the style differentiation, and the basal appendages of the anther begin to differentiate (Fig. 4d). The cells at the apex of the styles are rounded (Fig. 4d) and phenolic (Fig. 4e), and the proximal portion of the filaments turn adnate to the petals for postgenital union (Fig. 4e). The basal appendages of the anther elongate, positioning themselves below the pollen sacs and all organs enlarge (Fig. 4f). The apex of the styles remains free from each other, but just below this region, the styles become united by post‐genital fusion, forming the style head (Fig. 4g). The style head becomes larger, with a conical shape (Fig. 4g). The ovary turns pilose, and the nectary is flattened dorsoventrally with a laminar shape (Fig. 4g). All the organs enlarge again (Fig. 4h). At the pre‐anthesis stage, it is also possible to observe that the ventral region of the anthers, just above the insertion of the filaments, becomes indumented (Fig. 4g). The expansion of the filaments and styles surpasses the internal capacity of the floral bud, resulting in a tensioned configuration (Fig. 4h). In the distal region of the style head, phenolic cells are visible. (Fig. 4i). The anther becomes compartmentalized, with the pollen sacs concentrated in its distal portion, while the basal appendages of the anthers are still in the proximal portion (Fig. 4h–j). The dorsal part of the connective features sclerenchymatic cells immediately below the epidermal layer, nestled between the two abaxial pollen sacs and pollen grains are dispersed in monads (Fig. 4i). The proximal region of the style‐head exhibits a pentalobate form, with elongated secretory cells (Fig. 4h, j). Each lobe of this structure contacts the connective at the anthers, which displays a minor adaxial protrusion (Fig. 4j). A secretion is observed between the anthers and the style head spaces (Fig. 4j). It is also possible to observe that the connective region presents elongated cells that come into contact with the lobed portions of the style head, which has a star shape at this stage. This assembly of anthers and style heads collectively forms a gynostegium despite the continued release of pollen grains in monads.

### The mid and final developmental stages of the flower of *Asclepias curassavica*

**Fig. 5:**
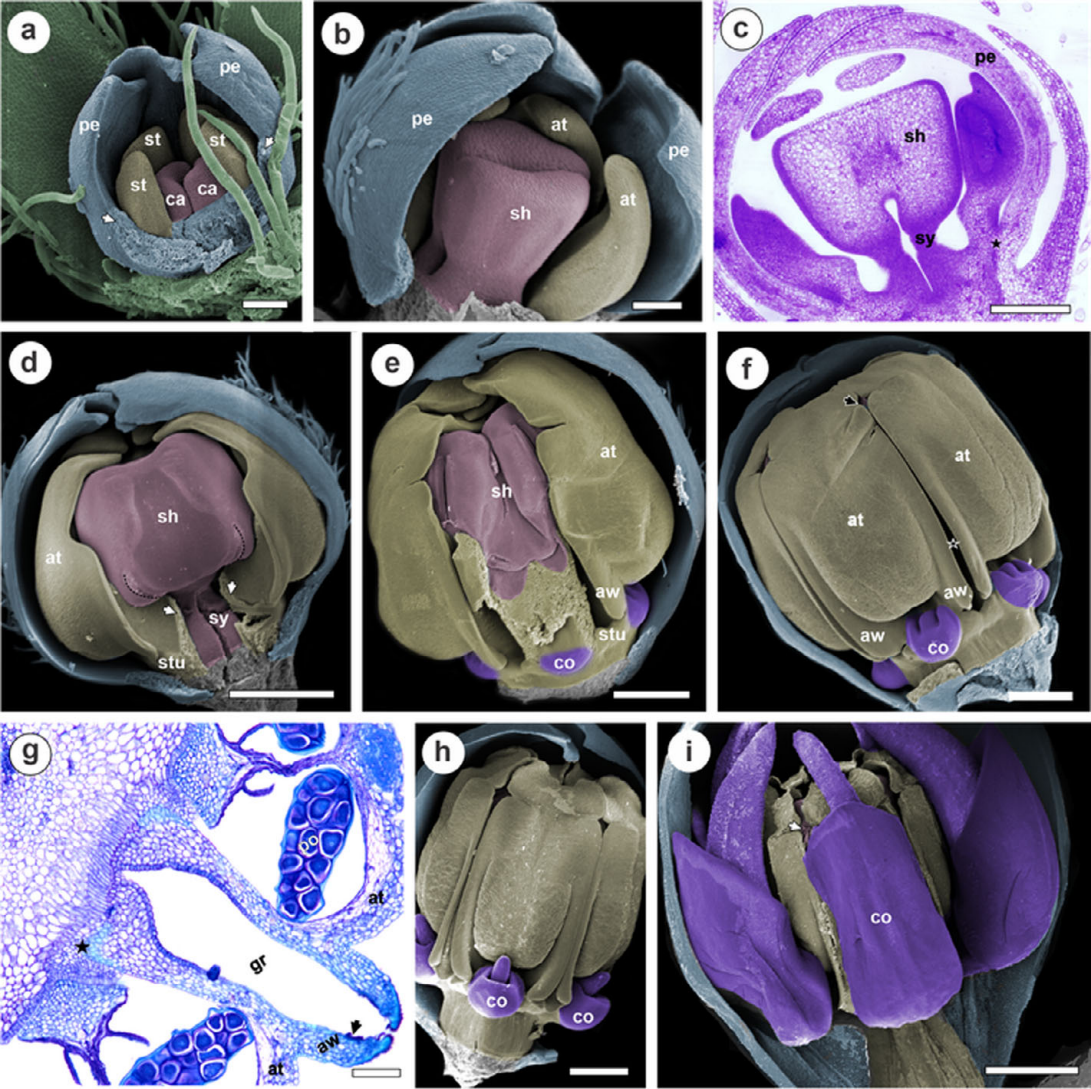
Mid and late developmental stages of the flower of *Asclepias curassavica* L. under light (c, g) and scanning electron microscopy (a, b, d–f, h, i). (a) Young floral bud with all whorls established, the base region of the fused corolla (white arrow) in a short tube. (b) The apical region of the differentiated carpels forms the style head, and the basal region differentiates into free ovaries. (c) Detail of the carpels united at the apex and free at the basis; overlap between filaments and petals basis (star). (d) The ventral region of the filaments, forming a staminal tube around the ovaries (white arrow), dashed lines on the style‐head indicate the scars left by the removed anther. (e) The constricted style head expands and folds around the anthers; the formation of coronas begins in the staminal tube region below the anthers. (f) Trilobed coronas at the beginning of development, with details on the anther wings of adjacent anthers forming the guide rails (star); a pollinarium corpuscule is visible (black arrow). (g) Adjacent anthers in detail, highlighting the anther wings with sclerenchymatic tissue, and the sclerenchyma region on the ventral side of the pollen sacs (star), secretory tissue in the apex of the anther wings (arrow). (h) The outer lobes of the corona unite and enclose the central lobe as a ring, the middle beginning elongate. (i) Pre‐anthesis bud. Calyx (green); corolla (blue); androecium (yellow), gynoecium (pink); coronas (purple) at: anther; aw: anther wings; ca: carpel; co: corona; fi: filament; gr: guide rails; ov: ovary; pe: petal; se: sepal; sh: style head; st: stamen; stu: staminal tube; sy: style. scales: A–B, G = 100 μm; C = 200 μm; D–F, H = 500 μm; I = 1 mm.

**Fig. 6:**
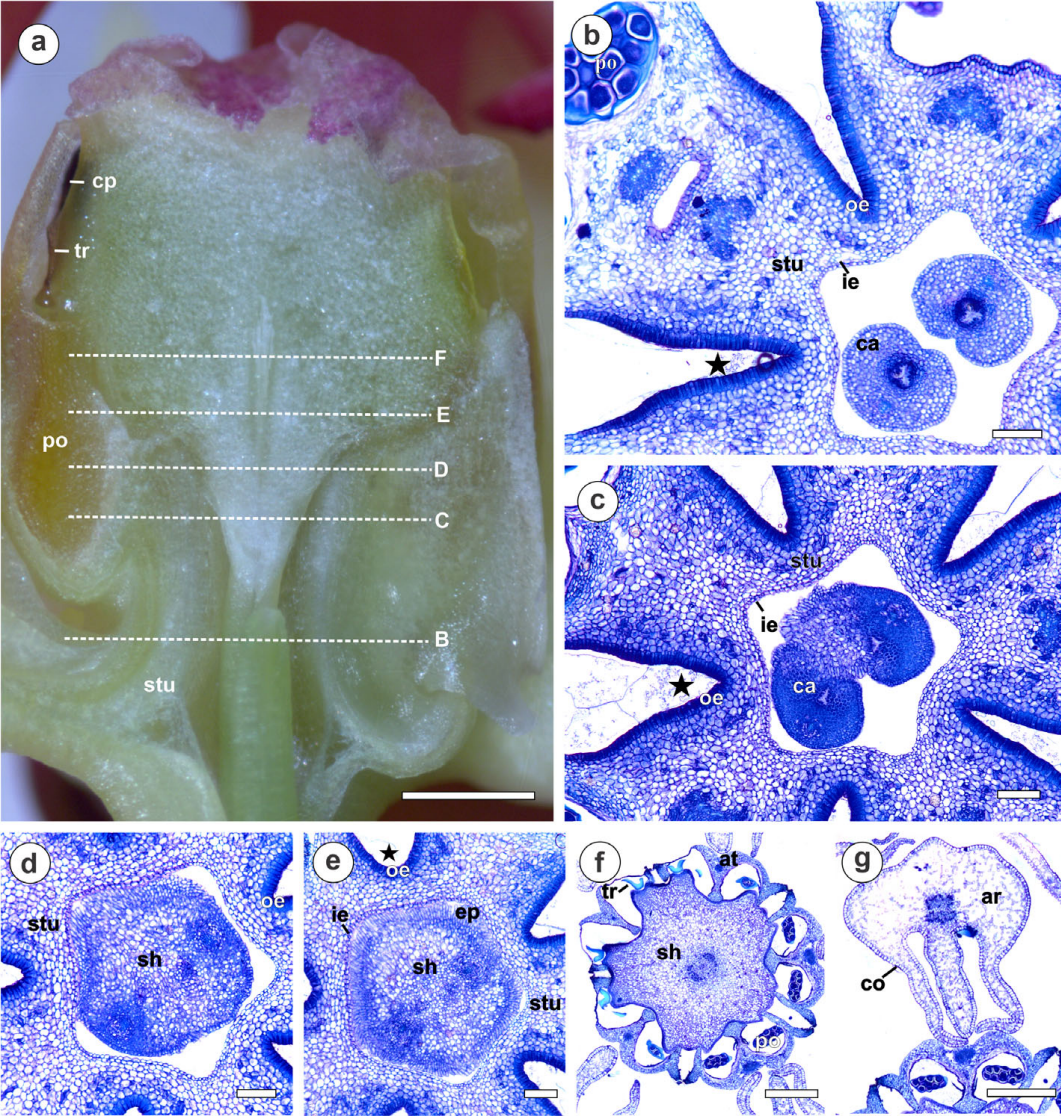
Union between the anther and style heads during the formation of the gynostegium in *Asclepias curassavica* L. and staminal changes observed under a stereomicroscope (longitudinal section) and light microscopy (transverse sections). (a) Pre‐anthesis bud highlighting the gynostegium with dotted lines locating the illustrated anatomical cuts. (b) Basal region of the anthers within the gynostegium, with secretory cells at the proximal guide rails; Carpels still free, enveloped by the staminal tube. (c) Region of carpel union. (d) Increasing the size of the carpel union. (e) Carpels completely occupy the region delimited by the inner epidermis of the staminal tube. (f) The complete union between the carpels and the staminal tube; at this stage, it is no longer possible to precisely delineate the boundaries between the anthers and the style head. (g) Staminal corona details. Star: guide rails; ar: aerenchyma; at: anther; ca: carpel; co: corona; cp: corpusculum; ep: epidermis; ie: inner epidermis; oe: outer epidermis; po: pollinia; sh: style head; st: stamen; stu: staminal tube; sy: style; tr: translator. scale: A = 0.8 mm; B–E = 100 μm; F = 500 μm.

Following the organ initiation in the four whorls, they undergo an enlargement phase during which a short union between the petals at the proximal region can be observed. In contrast, the majority of the petal extension remains free, and the apex of the carpels converges laterally (Fig. 5a), adopting a clavate form (Fig. 5b,c). This region is differentiating into a connate style head. At this stage, a short adnation between the filaments and the petals base occurs (Fig. 5c). However, they remain free from each other for almost their entire length (Fig. 5c). The stamens are composed of two main regions: the anther and the filament. The anther region is differentiated at the apex. The region that comprises the pollen sacs is positioned lower (Fig. 5d). The filaments of the stamens are united with each other, forming a staminal tube around the carpels (Fig. 5d). The style head meets completely on the lateral sides. It remains distinct on the distal side, assuming a clavate shape. The basal part of the carpels, however, remains free (Fig. 5b–d). The bases of the anthers join through adnation with the style head, oriented in the radial direction (Fig. 5d). The intricate folding and unique specializations of the anthers inherently limit the spatial availability for the style head, thereby compelling the style head to conform to the shape dictated by the anther's specializations (Fig. 5e). The anthers lateral margins project radially, forming the ‘anther wings’ (Fig. 5e–g). The meeting of anther wing from adjacent anthers forms a narrow space that extends throughout the length of the anthers, known as ‘guide rails’ (Fig. 5f,g). The projections that form the anther wings have lignified cells, and the lateral sides of the inner part maintain contact with the tissues of the style head (Fig. 5g). The region where the anther wings meet the adjacent anther wing has secretory cells. It is possible to observe secreted material around the structure (Fig. 5g). The anther apex becomes tapered and flattened, bending towards the style head. This bending exposes the structure that forms the translators of the pollinaria, providing access for pollinators (Fig. 5f,i). The areas of adnation between the stamens and the style head also increase, resulting in an almost complete connection between their organs, forming the gynostegium structure (Figs 5e and 6a–f). At this stage, it is possible to observe the inception of the coronas formed by staminal projections beneath the anthers (Fig. 5e). The staminal tube forms a concave structure opposite the guide rails (Fig. 5e–f, h). The coronas originate from a trilobate structure (Fig. 5f). The lateral lobes of the corona join to form a ring around the middle lobe, which in turn becomes more elongated than the rest of the structure (Fig. 5h). The coronas persist in elongating until they surpass the gynostegium in height (Fig. 5i). The two carpels only keep together in the distal portion of the styles, near the style head and the free portion of the styles are completely enclosed by the staminal tube (Fig. 6a). In the transverse section, it is possible to observe the internal epidermis of the staminal tube, which delineates the pentalobate space where the styles are inserted (Fig. 6b). At the basal portion of the anthers, where the pollen sacs are located, there is a union with the staminal tube, positioned more internally. Bordering this structure, it is possible to observe the outer epidermis, composed of juxtaposed cells in palisade with dense cytoplasm, indicating secretory activity in this region of the stamen epidermis (Fig. 6b–f). The styles, through the process of connation, unite to constitute the style head and subsequently expand their volume (Fig. 6c–f). As this structure nears the distal segment of the gynostegium, the burgeoning style head propels the staminal tube towards the periphery (Fig. 6e–f). The distal part of the gynostegium is predominantly composed of the style head, which surpasses the stamens in volume (Fig. 6f). At this juncture, the demarcations between the anthers and the style become indiscernible within a specific region, signifying complete adnation of these organs (Fig. 6a–f). The pollen grains are dispersed as pollinia that form on the pollen sacs (Fig. 6a,f). The coronas receive vascular supply and are filled with aerenchyma (Fig. 6g).

## DISCUSSION

It is widely accepted that numerous morphological changes were essential to the flowers in Apocynaceae to attain such advanced levels of synorganization. This intricate process led to the development of the gynostegium, which subsequently enabled the formation of pollinaria, within a complex pollination system that involves both the tissues of the style head and those of the anthers (Endress 2016; Demarco 2017a, 2017b; Ollerton *et al*. 2019). Our results illustrate some of these steps in the floral development of Apocynaceae flowers and shed light on changes in the corolla tube and in the stamen.

### Comparative floral development in different flower types of Apocynaceae

**Fig. 7:**
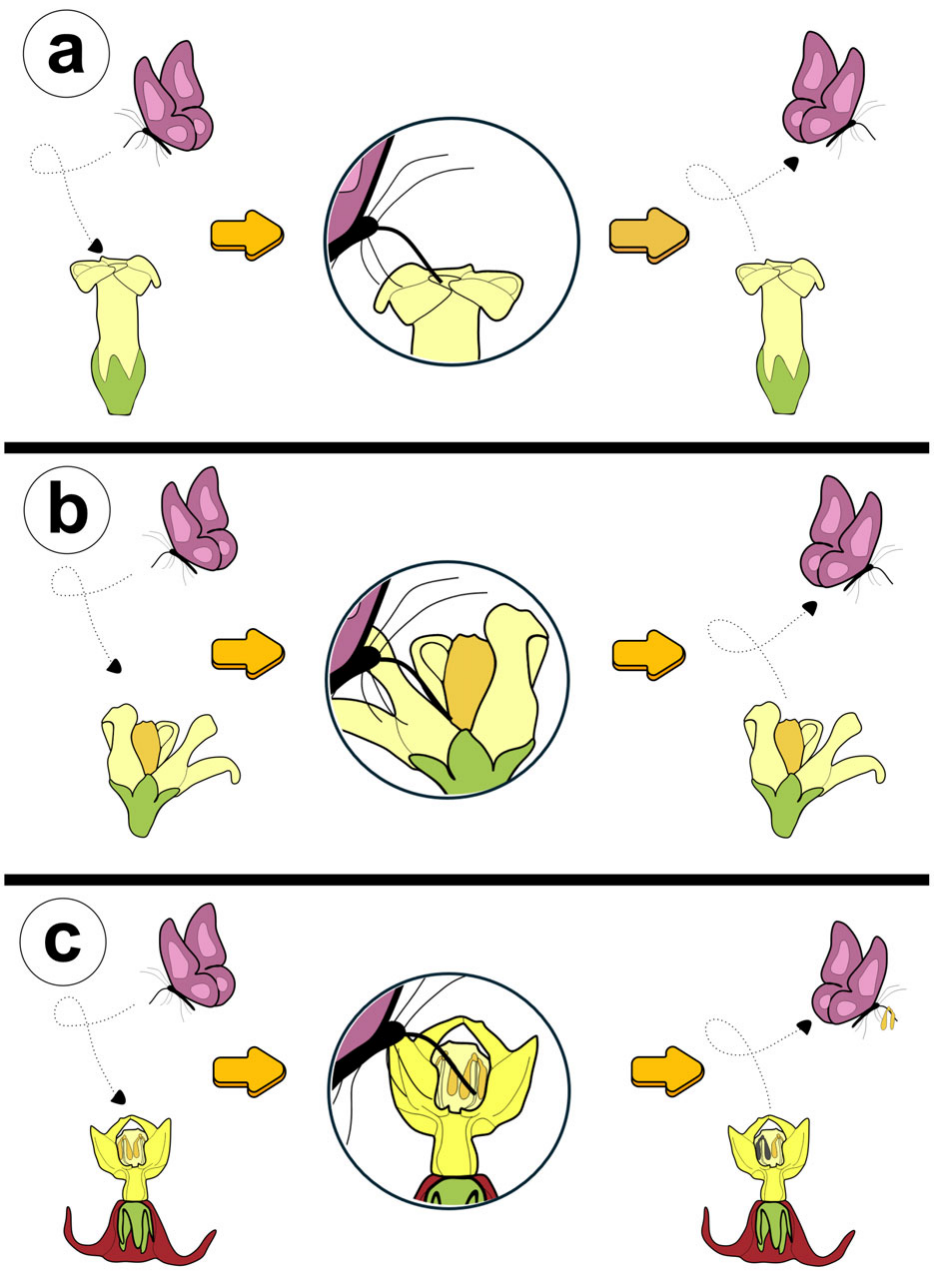
Schematic representation showing the access of the pollinator proboscis to the flower. (a) *Aspidosperma australe* Mull. Arg. (b) *Forsteronia glabrescens* Mull. Arg. (c) *Asclepias curassavica* L.

The floral organs generally arise as independent primordia in the analysed species. Accordingly, organ union is interpreted as predominantly postgenital, although the developmental pattern observed for corolla tube formation in *A. curassavica* suggests a distinct condition that is discussed below. In particular, its fusion process, giving rise to the gynostegium in Apocynaceae through postgenital fusion, is widely accepted in the literature (Greyson 1994; Simões *et al*. 2007; Kunze & Wanntorp 2008; Endress 2016; Heiduk *et al*. 2020). We focused our analyses on comparing how and when changes in the petals, stamens and carpels occur through the processive changes that culminated in the gynostegium development, evaluating (i) formation of the corolla tube; (ii) epipetalous stamens; (iii) stamen modifications; (iv) secretory tissue and nectar; and (v) proboscis targeting flowers (Fig. 7).

Concerning corolla tube formation, the fusion of petals into a tube is a defining feature of Apocynaceae (Fallen 1986; Endress & Bruyns 2000; Kunze 2005; Endress 2016; Fishbein *et al*. 2018). In lamiids, corolla tube development has traditionally been interpreted as congenital sympetaly resulting from zonal growth during early floral ontogeny (Erbar & Leins 1996; Ronse De Craene 2010). However, floral organ fusion may result from distinct developmental processes. Congenital fusion involves the formation of a compound structure through a common basal meristematic region, whereas postgenital fusion occurs when initially separate primordia later come into epidermal contact and adhere. Although these processes may produce morphologically similar mature structures, they are ontogenetically distinct (Verbeke 1992).

In Apocynaceae s.s., postgenital fusion has frequently been described as contributing to corolla tube formation (Sennblad *et al*. 1998; Kunze 2005), particularly in rauvolfioid and apocynoid grades. Nevertheless, this is not the only developmental pathway documented within the family. In Asclepiadoideae, corolla tube formation has often been interpreted as congenital across most genera (Kunze 2005), although exceptions occur in less derived tribes such as Fockeeae and Marsdenieae. Erbar & Leins (1996) noted that corolla development in Asclepiadoideae may represent an intermediate condition between early and late sympetaly, in which petal primordia arise separately but their bases rapidly connect around the time of stamen initiation. Importantly, early and late sympetaly describe variation within congenital sympetaly rather than a contrast with postgenital fusion. Similarly, Verbeke (1992) reported that, in some Apocynaceae, the basal portion of the tube may result from zonal growth, whereas the upper portion involves postgenital adhesion between petal bases, highlighting developmental diversity within the family. In *Ceropegia sandersonii* Decne. ex Hook., for example, petal primordia arise freely, yet the lower portion of the corolla tube has been interpreted as congenitally formed (Heiduk *et al*. 2020). In the species analysed here, petal primordia arise as independent flanks, and no continuous basal meristematic ring was observed during early development. In *A. australe* and *Forsteronia glabrescens*, the corolla tube becomes evident only after stamen initiation, when adjacent petals progressively come into contact, a pattern consistent with postgenital fusion. In *A. curassavica*, however, tube formation occurs at an earlier developmental stage, reflecting a shorter plastochron between petal initiation and tube formation. Despite the initial independence of petal primordia, the ontogenetic evidence in this species does not clearly demonstrate epidermal continuity between adjacent petals during tube formation, as would be expected in typical postgenital fusion. Furthermore, additional criteria often associated with congenital fusion, such as the presence of an intercalary meristem or shared vascular bundles, were not observed as supported by recent vascular analyses in the same species, which demonstrate the absence of shared vascular bundles among petals (Alves *et al*. 2026). Taken together, these observations make the interpretation of corolla tube formation in *A. curassavica* less straightforward. In the absence of clear evidence for postgenital adhesion, this condition is better discussed in light of previous studies in Asclepiadoideae, where corolla tube formation has frequently been interpreted as congenital (Erbar & Leins 1996; Kunze 2005). Thus, while postgenital processes appear to predominate in the taxa examined here, corolla tube formation in *A. curassavica* may represent a distinct developmental condition, highlighting the diversity of ontogenetic pathways underlying sympetaly in Apocynaceae. The shorter plastochron observed in *A. curassavica* may help explain the difficulty in classifying corolla tube origin in the former Asclepiadaceae, as highlighted by Erbar & Leins (1996). The plastochron between the emergence of the petal primordium and the formation of the corolla tube can be a determining point in the tube's length, too. This interval is sufficiently extensive to dispel any uncertainties regarding the postgenital origin of the corolla tube, particularly within the early divergent Apocynaceae taxa. Our results corroborated this understanding. In *A. australe*, for example, the fusion between the bases of the petals occurs concomitantly with the differentiation of the carpel styles, and the corolla tube is relatively longer than in the other species analysed. In *F. glabrescens* and *A. curassavica*, the formation of the corolla tube seems to occur after the initiation of the carpels, at a less advanced stage and they are shorter than that of the *Aspidosperma* flower. However, among the species analysed here, *A. curassavica* has the smallest corolla tube and is likely the first to form. In addition to the shorter corolla tube in *F. glabrescens* and *A. curassavica* compared to *A. australe*, we also observed that the anthers in *F. glabrescens* and *A. curassavica* are positioned at the same height as the style head. In contrast, in *A. australe*, the anthers are placed above the style head. Although our data indicates a potential correlation between the plastochron of corolla tube formation and the corolla tube size, further studies encompassing a broader range of taxa within the family are necessary to substantiate these findings. Transitions among corolla forms were examined by Fishbein *et al*. (2018) in a broad character evolution analysis. The ancestral corolla of Apocynaceae is most likely salverform, whereas in the APSA clade, it is inferred to be infundibuliform. For Periplocoideae, Secamonoideae and Asclepiadoideae, the ancestral state remains ambiguous, although transitions between tubular and urceolate corollas are more likely, with rotate forms emerging at deeper nodes of Asclepiadoideae. Another aspect discussed in Fishbein *et al*. (2018) study concerns exerted stamens, indicating that, although still uncertain, it is more likely that stamens inserted within the corolla tube originated in the deeper nodes of Asclepiadoideae, with subsequent transitions towards partially exerted and fully exerted forms across the family. However, for the ancestor of the Secamonoideae + Asclepiadoideae clade, exerted anthers are considered the characteristic condition. Our ontogenetic evidence is consistent with these results, indicating that the corolla tube becomes progressively shorter and the stamens become increasingly detached from the corolla.

Considering the attachment of stamens to the corolla tube (‘epipetalous stamens’), our findings indicate that, within the analysed species of the APSA clade, the association between the stamens and the corolla tube diminishes. We could verify that by the variation in the degree of filament insertion, ranging from complete integration within the tube exemplified by *A. australe* (rauvolfioid grade) to minimal insertion from the corolla tube, in *F. glabrescens* (apocynoid grade), and *A. curassavica* (Asclepiadoideae). Epipetalous stamens are a common trait among asterids (Erbar 1991; Erbar & Leins 1996; Ronse De Craene 2010) and are commonly reported in Apocynaceae s.s. (Fallen 1986; Endress & Bruyns 2000; Kunze 2005; Fishbein *et al*. 2018). Our findings reveal filaments entirely inserted into the corolla tube in *A. australe*, with anthers in the distal section of the tube, immediately beneath the corolla lobes. In contrast, in *F. glabrescens* and *A. curassavica*, the filaments are attached to the corolla tube solely at the proximal region, remaining unattached for most of their length. The whole fertile anthers positioned in the upper part of the corolla tube, surpassing the style head, is a characteristic of plants with early divergence within Apocynaceae, as observed in most species of rauvolfioid. Conversely, within the apocynoid grade, the anthers are known for their insertion into the corolla tube at the median part, aligning parallel to the style head (Fallen 1986), with pollen sacs restricted to the distal part of the anthers, positioned above the style head. Later, ‘stamen insertion’ was employed as a distinguishing character state to rauvolfioid and apocynoid grades, also considering Asclepiadoideae, which is described as inserted at the basal region of the corolla tube or around the ovaries, directly on the floral receptacle (Endress & Bruyns 2000). In addition to the diminished association between the filaments and the corolla tube, our findings also reveal that even in species where the filaments appear entirely free from the corolla tube in the mature flower, brief epipetaly can still be observed at the onset of floral development, as demonstrated for *A. curassavica*. This suggests that, despite the progressive reduction of the extension of the corolla and filaments union, it remains present at the initial stages of floral development in the more recent groups.

Another aspect that deserves attention is the stamens modifications, including the plasticity and sclerenchymatic cells of the anthers, as well as secretory activity (as discussed below), through Apocynaceae. The anthers range from relatively simple and free anthers, without specializations, with four pollen sacs as in most angiosperms, to anthers adnate to the style head, with sclerenchymatic cells in part of them, a specialized connective and a reduced number of pollen sacs per anther in Asclepiadoideae. The insertion of the filaments could be dorsal with a simple connective (as in *Aspidosperma*), ventral with hair pads in connective (Fallen 1986 and here in *Forsteronia*) or they can form a staminal tube as in the asclepiads (*i.e., A. curassavica*). The dorsal sclerenchymatous portion of the anthers, located around the connective and extending below the thecae to form two lateral wings, was designated by Wanntorp (1988) as the ‘peltate connective’. Subsequently, Pichon (1948) regarded this tissue as a specialization of the anthers and proposed the term ‘retinacle’ also to denote the sterile, non‐sclerenchymatous portion on the ventral side of the connective, which adheres or remains adjacent to the style head, forming the gynostegium. Later, the term ‘retinacle’ was further specified as ‘staminal retinacle’ to avoid confusion with the homonymous structure of the pollinarium in Orchidaceae (Simões *et al*. 2007). Sclerenchyma in the stamens is not a very common feature among angiosperms, and when it occurs, it has arisen independently in response to distinct selective pressures (Schmid 1972). Two roles can be attributed to the hairs that develop on the dorsal portion of the connectives: assisting in the adhesion between the anthers and the style head, as well as facilitating the pollination process by functioning as a ‘brush’ that helps remove pollen grains from the proboscides of insects (Pichon 1948; Fallen 1986). Our results indicate that anther specializations are still present in more recent lineages, as observed in *A. curassavica* (Asclepiadoideae). In this species, sclerenchymatic cells develop not only in the dorsal portion of the connective but also along the lateral sides, in the anther wings. These structures contribute to the delimitation of the guide rails in this group. Beyond these modifications, the number of pollen sacs per anther also decreases, going from four to two pollen sacs per anther in Asclepiadoideae species. Endress (2016) suggests the link between the formation of a sterile and reinforced tissue on the flanks of the anthers of the Asclepiadoideae (guide rails) to the loss of the two dorsal pollen sacs in the group. However, we could not apply this suggestion to the apocynoid grade species, where sclerenchymatic cells in anthers are widely reported. Further investigation considering also Secamonoideae and Periplocoideae flowers that have four pollen sacs in their anthers will be interesting for understanding the relationship between the reduction of pollen sacs and the development of sclerenchymatic wings.

Considering secretory activity, our findings indicate secretory activity in the basal portion of the style head in *F. glabrescens*, as evidenced by hairs, as was previously reported by Fallen (1986) and by Simões *et al*. (2007). Similarly, the stamen filament tube in *A. curassavica* appears to exhibit a secretory function, particularly in the region of the guide rails. Demarco (2017b) investigated these secretory regions in the anthers of Asclepiadoideae species, demonstrating that both the internal region of the guide rails and the anther wing region exhibit secretory activity. He linked this secretion to the pollination process, in which the secretion from the guide rail region aids in the adhesion of the pollinator proboscis to the pollinarium and facilitates the sliding of the pollinia into the flower when the pollinator removes the clip from the style head. Similarly, the relationship between the secretions produced by the style head in *Forsteronia* L. and *Apocynum* L. (Fallen 1986; Endress 2016) and the tribe Mesechiteae (Simões *et al*. 2007) had already been associated with the pollination process, facilitating the adhesion of pollen grains to the pollinator proboscis. Integrating our results with the existing literature, we suggest that the secretions aiding in the complex pollination process observed in the family migrated from the gynoecium in species of the apocynoid grade to the androecium in Asclepiadoideae.

Likewise, we can also observe that the role of nectar production appears to migrate from the gynoecium whorl to the androecium. In groups that diverge early in the family, such as the tribe Aspidospermateae, the presence of nectaries is not attributed; however, nectariferous tissue around the ovary has been reported for *A. quebracho‐blanco* (Lin & Bernardello 1999) and *A. australe* (Russi *et al*. 2025). Nectaries at the base of the ovary are widely reported for the rauvolfioid and apocynoid grades (Woodson & Moore 1938; Fallen 1986; Endress & Bruyns 2000; Endress *et al*. 2014). In Asclepiadoideae, however, nectaries are no longer associated with the gynoecium. Demarco (2017a, 2017b) attributes the role in nectar production to tissues linked to the androecium, such as staminal coronas and staminal tube tissues. The species in this study illustrate the transition of nectaries from the gynoecium to the androecium in these three types of flowers. In *A. australe* (rauvolfioid), nectaries are not morphologically distinguishable, but the presence of a nectariferous tissue at the base of the ovary has been reported for the species and the genus (Lin & Bernardello 1999; Russi *et al*. 2025). In *F. glabrescens* (apocynoid), we can observe five nectaries around the ovary, and in *A. curassavica*, secretory structures are observed associated with the parts of the anthers, also without any morphologically distinct structure (Demarco 2017a, 2017b).

### Adnation between the anthers and the style head forming the gynostegium

Our results reveal three distinct stages of approximation between anthers and the style head. In *A. australe*, their closeness results solely from the narrow floral tube with an inside upper indument, as anthers and style head remain completely free. In *F. glabrescens*, elongated cells of the style head interlace with ventral anther cells (staminal connective), with the region also covered by secretions from the style head. In *A. curassavica*, two successive adnation events occur: filaments fuse to form a staminal tube, followed by the proximal anthers becoming adnate to the style head, both through tissue fusion. The three stages of stamen–style head union are well established in the literature of the family and have long been recognized as important morphological characters for distinguishing groups within Apocynaceae (Pichon 1950; Fallen 1986; Endress & Bruyns 2000; Simões *et al*. 2007).

The union between anthers and the style head, occurring through hairs or elongated epidermal cells and hairs of the ventral side of the anthers, or by secretion, had already been described in detail by Simões *et al*. (2007) for the tribe Mesechiteae, and by Fallen (1986) for Apocynoid groups as Echitoideae. In *Forsteronia* and *Apocynum*, this secretion may occur in the form of small gummy platelets that assist in capturing pollen grains by the proboscis of pollinators, an innovation regarded as a precursor of the translator in the pollinia of more derived groups (Demeter 1922; Fallen 1986; Nilsson *et al*. 1993; Endress 2016).

Regarding the union between the anthers and the style head, whether by secretion or by postgenital fusion forming the gynostegium, it is likely that this feature first appeared in the most recent common ancestor of the APSA clade (Wanntorp 1988; Judd *et al*. 1994; Livshultz *et al*. 2007; Fishbein *et al*. 2018), and subsequently regressed on two independent occasions, in *Holarrhena* R.Br. and *Spirolobium* Baill. (Malouetieae). Fishbein *et al*. (2018) emphasize that the structural rigidity of gynostegium formation across the phylogeny contrasts with the lability of anther position relative to the style head within the rauvolfioid grade.

About the timing of adnation between the anthers and the style head during development, our results indicate that in *F. glabrescens* it occurs only at the final stages of floral development, when the style head is already fully differentiated and the secretory function established. In *A. curassavica*, by contrast, adnation takes place at earlier stages. This adnation suggests that, as the trait becomes established, its expression shifts to earlier phases of floral development. Most studies on floral heterochrony emphasize scenarios in which organs are already formed and timing adjustments mainly yield variation in size and shape (Box & Glover 2010; Buendía‐Monreal & Gillmor 2018; Ronse De Craene 2024). In contrast, our results indicate an earlier onset (predisplacement) of the adnation that forms the gynostegium, such that, as the trait becomes established within the family, its expression shifts to earlier ontogenetic stages, explaining the observed gradient in the degree of adnation.

### Different ways to conduct the pollinator proboscis into the flower

Our results show that in *A. australe*, the corolla tube surpasses the anthers in height and, besides its inside indumentum, keeps the anthers close to each other and restricts the pollinator access to the flower centre, whereas in *F. glabrescens*, the corolla tube is restricted to the proximal region and, in the anthesis, the gynostegium structure is wholly exposed to the environment and to the pollinator (Fig. 7a,b). Therefore, in addition to guiding the proboscis during pollination, the stamens require support for the pollination to occur. The sclerenchymatic tissue added to the anthers, the adnation to the style head and the position of the stamen filaments can perform this role in *F. glabrescens* flowers or other flowers where the corolla tube is shorter and less constricted than in rauvolfioids (and some apocynoid) species. Sclerenchymatic tissues primarily provide mechanical or structural support (Evert 2006), consistent with the supportive role of the sclerenchymatic anthers in apocynoid species. In Apocynaceae, the occurrence of sclerenchyma is a functional coadaptation that refines pollination mechanisms (Fallen 1986). In the most recent diversified species, such as those in the Asclepiadoideae subfamily, the filaments are connate, forming a staminal tube. In this group, support is not limited to the anthers; it extends to the entire androecium (Fig. 7c). The staminal tube, which is free or mostly free of the petals, can help keep the gynostegium elevated. Kunze (1991) emphasized adaptation devices essential to the pollination process, such as the filament tube, which is filled with nectar during anthesis, the anther slit, through which the pollinator proboscis passes and the coronas which help guide the pollinator during pollination. Demarco (2017b) added the production of nectar by the region of the guide rails. In *A. curassavica*, we could also observe in the anatomical section that the inner part of the coronas is filled with aerenchyma (Fig. 6g). This adaptation can provide structural support to the gynostegium, as the air‐filled structure makes the coronas lighter and confers stability to the gynostegium together with the filament tube and the anthers.

In earlier divergent species, the narrow corolla tube and hairs ensure that the anthers are close to the style head, which guides the pollinator proboscis during pollination (Fallen 1986; Fishbein *et al*. 2018). Nevertheless, as discussed in the previous section, the anthers detached from the corolla tube and became strongly attached to the style head in most derived taxa. Consequently, the function of guiding the pollinator proboscis was transferred from the corolla tube to the androecium. It had previously been suggested that the gynostegium replaces the corolla in guiding the pollinator during pollen transfer (Fallen 1986). That may contribute to the irreversibility of the gynostegium within the family, reflecting selection driven by this functional shift (Fishbein *et al*. 2018). The sclerenchyma specializations in the proximal portion, along with the hairs that unite the anthers to the style head, are the adaptations for this functional substitution (Fallen 1986). Our findings agree with these assertions, and we would like to highlight another structural role transferred to the androecium. In summary, our analyses reveal that, in addition to guiding pollination, the mechanical support function is also shifted from the corolla to the androecium, which is essential for pollination. As the floral resource is nectar, the structures/tissues that produce nectar are no longer located on the wall of the gynoecium or in surrounding nectaries and are now produced from the stamens. The same occurs for the function of secreting substances to facilitate the pollen transfer, which changes from the style head to the stamen.

## CONCLUSIONS

Our analysis provides, for the first time, a comparative account of floral development across representative flower types of Apocynaceae and highlights the floral modifications that culminated in the formation of the gynostegium in the APSA clade, emphasizing the role of functional transfer in these processes. We show that the plastochron between petal initiation and corolla tube development strongly influences how corolla tube origin is interpreted in the family and appears to be related to floral tube length. In the species examined here, corolla tube development is generally consistent with postgenital processes, although the developmental pattern observed in *A. curassavica* suggests that this interpretation may not be uniform across Apocynaceae. We also observed that stamen epipetaly tends to be reduced in more recently diverged groups within the family, as seen in *Forsteronia* and especially in *A. curassavica*.

Although the union between stamens and the style head is fundamental to the origin of the gynostegium, a key structure in the evolution of the highly specialized pollination system of Apocynaceae, our results show that other floral modifications were also necessary and played an important role in its assembly. Comparative ontogenetic evidence indicates that the main changes associated with gynostegium evolution involve corolla tube development, shifts in the degree of stamen epipetaly and specializations of the stamens, particularly of the anthers, that enabled their adnation to the style head. Among these, stamen specializations emerge as especially important in the progressive establishment of the gynostegium across the family, including the development of the staminal connective, the sclerenchymatic tissues of the anthers and the staminal tube initiated by the free portion of the filaments. These modifications provide mechanical support to the gynostegium and assist pollination by guiding the pollinator proboscis, as observed in *Forsteronia* and *Asclepias*. In this evolutionary context, our results support the earlier studies showing that the role of guiding and supporting the pollinator proboscis, initially performed by the corolla tube in early‐diverging groups, appears to have been progressively transferred to the androecium.

Our results also corroborate a broader pattern of functional transfer of secretory activity from the gynoecium to the androecium across the family. This is evidenced by the shift in secretory roles from the style head and ovary‐associated nectaries to anther tissues, as demonstrated in *A. curassavica*. The transfer of functions related to attraction (nectar secretion), pollinator guidance and facilitation of pollination, mediated by morphological and histological specializations of the androecium, appears to have been essential for the origin of the gynostegium and for the establishment of the highly complex pollination system found in Apocynaceae.

Because our conclusions are based on a single species per group, broader taxonomic sampling will be essential to refine our understanding of floral ontogeny in the family. Future studies including the subfamilies not sampled here due to geographical constraints (*Periplocoideae* and *Secamonoideae*) will be particularly important for evaluating the diversity of developmental pathways involved in corolla tube development and for providing new insights into the origin of the gynostegium in Apocynaceae.

## AUTHOR CONTRIBUTIONS

DMA: Draft of the manuscript, collection of material and analysis of data; LSS: Analysis of data and contributions on the draft of the manuscript; IK and JLSM: Work supervision and contribution on the draft of the manuscript. All authors reviewed the results and approved the final version of the manuscript.

## Data Availability

Data sharing not applicable to this article as no datasets were generated or analysed during the current study.
